# Bactericidal Permeability-Increasing Proteins Shape Host-Microbe Interactions

**DOI:** 10.1128/mBio.00040-17

**Published:** 2017-04-04

**Authors:** Fangmin Chen, Benjamin C. Krasity, Suzanne M. Peyer, Sabrina Koehler, Edward G. Ruby, Xiaoping Zhang, Margaret J. McFall-Ngai

**Affiliations:** a Department of Microbiology, College of Resources, Sichuan Agricultural University, Chengdu, Sichuan, People’s Republic of China; b Kewalo Marine Laboratory, Pacific Biosciences Research Center, School of Ocean and Earth Science and Technology, University of Hawaii at Manoa, Honolulu, Hawaii, USA; c Department of Medical Microbiology and Immunology, University of Wisconsin—Madison, Madison, Wisconsin, USA; Palo Alto Health Care System

**Keywords:** *Vibrio fischeri*, antimicrobial peptides, bioinformatics, confocal microscopy, symbiosis

## Abstract

We characterized bactericidal permeability-increasing proteins (BPIs) of the squid* Euprymna scolopes*, EsBPI2 and EsBPI4. They have molecular characteristics typical of other animal BPIs, are closely related to one another, and nest phylogenetically among invertebrate BPIs. Purified EsBPIs had antimicrobial activity against the squid’s symbiont, *Vibrio fischeri*, which colonizes light organ crypt epithelia. Activity of both proteins was abrogated by heat treatment and coincubation with specific antibodies. Pretreatment under acidic conditions similar to those during symbiosis initiation rendered *V. fischeri* more resistant to the antimicrobial activity of the proteins. Immunocytochemistry localized EsBPIs to the symbiotic organ and other epithelial surfaces interacting with ambient seawater. The proteins differed in intracellular distribution. Further, whereas EsBPI4 was restricted to epithelia, EsBPI2 also occurred in blood and in a transient juvenile organ that mediates hatching. The data provide evidence that these BPIs play different defensive roles early in the life of *E. scolopes*, modulating interactions with the symbiont.

## IMPORTANCE

This study describes new functions for bactericidal permeability-increasing proteins (BPIs), members of the lipopolysaccharide-binding protein (LBP)/BPI protein family. The data provide evidence that these proteins play a dual role in the modulation of symbiotic bacteria. In the squid-vibrio model, these proteins both control the symbiont populations in the light organ tissues where symbiont cells occur in dense monoculture and, concomitantly, inhibit the symbiont from colonizing other epithelial surfaces of the animal.

## INTRODUCTION

Among the pattern recognition receptors (PRRs) that mediate host responses to biotic stimuli are those that have been conserved across evolution, including the members of the lipopolysaccharide (LPS)-binding protein (LBP) and bactericidal permeability-increasing protein (BPI) family (1, 2). LBPs and BPIs are secreted, and they recognize and bind the LPS of Gram-negative bacteria (3). LBP typically presents LPS to host surface receptors, an activity that ultimately induces changes in host gene expression; in contrast, the BPIs are bactericidal, binding to the surface and compromising the integrity of the bacterial cell envelope. Although these two functionally distinct classes of proteins are in the same family and share a characteristic molecular architecture, they have diverged considerably over evolution; even the isoforms of BPIs within a single species typically have a sequence similarity of only 40 to 50% (1, 4, 5).

Despite the differences between isoforms, the bactericidal activity of the BPIs (6), reports of their protective functions in mammalian epithelial tissues (7), and the antimicrobial activity of purified isoforms from aquatic animals (8, 9) have all suggested that these proteins play a key role in modulating the interactions of marine invertebrates with environmental microbes, which occur at ~10^6^/ml of seawater (10, 11). Mollusks (snails, clams, squid, and their relatives) have an open, dynamic body plan, wherein seawater is drawn across internal organs to ventilate the tissues. This body plan demands robust mechanisms to prevent uncontrolled colonization of these surfaces by bacterioplankton.

Analyses of the genomes and transcriptomes of mollusks have revealed encoded and expressed antimicrobial peptides and proteins in those epithelial surfaces that are likely to actively shape the communities of microbes with which the animals associate (1, 8, 12). Analyses of transcriptomic databases constructed from the tissues of the Hawaiian bobtail squid, *Euprymna  scolopes*, have revealed that four host proteins in the LBP/BPI family (initially named LBP/BPI 1 to 4) are expressed (2, 13). Thus far, the biochemical characterization of these proteins has suggested the presence of one LBP (*E.  scolopes* LPS-binding protein 1 [EsLBP1], pI ~6.9; [14]) and three BPIs (*E.  scolopes* permeability-increasing protein 2 [EsBPI2] through EsBPI4; pI > 9.0) (2, 13). Of particular interest were EsBPI2 and EsBPI4 (EsBPI2/4), as these two isoforms are unusually similar in sequence, and preliminary data not only suggested their regulation during the onset of symbiosis between the host’s light-emitting organ and its luminous symbiont, *Vibrio fischeri*, but also showed expression of these proteins in a variety of other tissues. In this study, we sought to explore the roles of EsBPI2 and EsBPI4 during early postembryonic development of the juvenile squid. The data presented here provide evidence that EsBPI2 and EsBPI4 have dual roles: (i) modulating activity of *V. fischeri* in the light organ and (ii) protecting against fouling of other epithelial surfaces by *V. fischeri*.

## RESULTS

### EsBPI2/4 are typical animal BPIs, both biochemically and phylogenetically.

**FIG 1:**
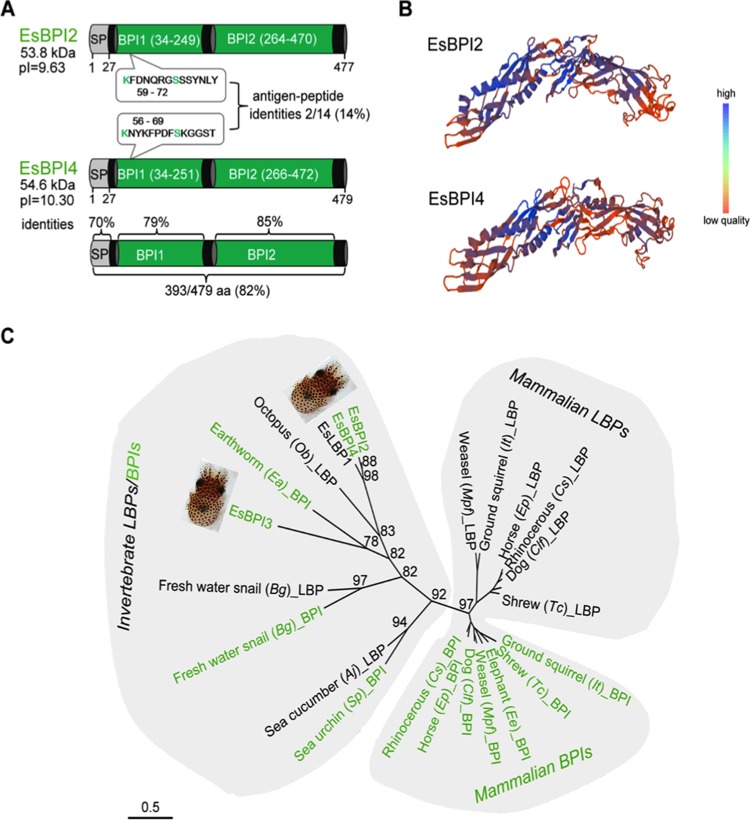
Characteristics of the EsBPI2 and EsBPI4. (A) Domain structure with full-sequence and domain alignment (SP, signal peptide; BPI1 and BPI2, bactericidal permeability-increasing domains). The boxes show the locations and sequences of the variable regions used for production of antibodies to EsBPI2 and EsBPI4. aa, amino acids; pI, isoelectric point; kDa, kilodaltons. (B) Model of EsBPI2/4 showing the characteristic “boomerang” shape predicted from human BPI templates (SWISS-MODEL template ID 1ewf.1.A and 1bp1.1.A, respectively). The color gradient indicates the structure quality by local QMEAN4 score (46). (C) An unrooted LBP/BPI phylogenetic tree produced by the maximum likelihood method (see Table S2 in the supplemental material for the species used and associated accession numbers). The numbers are aLRT values, which approximate likelihood ratio test scores (48) as percentages, and represent the level of confidence associated with the branch at a given node. The bar shows branch length distances as the number of amino acid substitutions per site.

EsBPI2 and EsBPI4 characteristics were predicted on the basis of the amino acid sequences derived from each transcript. EsBPI2/4 share with other BPIs the two-BPI domain structure, the presence of a signal sequence, and a very basic pI (Fig. 1A). They also share the predicted LPS-binding domain and conserved cysteines, which are involved in the formation of disulfide bonds critical for function of the LBP/BPI proteins (see Fig. S1 in the supplemental material). The protein sequence identity of EsBPI2 and EsBPI4 is 82% (see Table S1 for identity values and accession numbers); the nucleic acid sequences are 87% identical for both the open reading frame (1,262/1,443) and the complete transcript (1,489/1,714). The high overall protein and nucleic acid sequence similarity of EsBPI2 and EsBPI4 is unusual for BPI isoforms within an individual animal species (1, 4, 5). Where they differ, EsBPI4 disproportionately substitutes basic amino acids, although no evidence for positive selection was detected (see Materials and Methods). Only a few highly divergent regions were identified between the two proteins; a set of these regions was used for production of synthetic peptides used in antibody production (Fig. 1A). Both proteins are predicted to have the typical “boomerang-like” BPI structure (Fig. 1B).

10.1128/mBio.00040-17.2 FIG S1  Alignment of full-length sequences of EsBPI2 and EsBPI4 with one another and with EsLBP1 and human BPI (hBPI). Solid boxes depict regions corresponding to (i) residues 42 to 48 of human BPI, including conserved basic residues at positions 42 and 48 of human BPI and (ii) residues 92 to 99 of hBPI, including conserved basic residues at positions 92, 95, and 99 of hBPI (4). Black arrowheads indicate cysteines thought to correspond to the conserved disulfide bond of LBP/BPI proteins. The red arrow indicates the typical starting point for numbering of hBPI, which excludes the signal sequence (4). Basic residues (Arg and Lys) are shown in red, and acidic residues (Glu and Asp) are shown in blue. Download FIG S1, TIF file, 14.1 MB. Copyright © 2017 Chen et al. 2017 Chen et al. This content is distributed under the terms of the Creative Commons Attribution 4.0 International license.

10.1128/mBio.00040-17.6 TABLE S1  Protein sequence alignments and accession numbers. Download TABLE S1, DOCX file, 0.02 MB. Copyright © 2017 Chen et al. 2017 Chen et al. This content is distributed under the terms of the Creative Commons Attribution 4.0 International license.

Our phylogenetic treatment of select members of the LBP/BPI family was intended to show relationships between the *E. scolopes* isoforms and their relationships with other animal groups. A more in-depth phylogenetic analysis among members of this protein family across the animal kingdom has recently been performed (1). EsBPI2 and EsBPI4 are most closely related to one another (Fig. 1C). Their high sequence identity suggests that they diverged through recent gene duplication. Together, these two proteins are most closely related to EsLBP1, not the other EsBPI, EsBPI3. Thus, unlike the mammalian proteins, in which all LBPs group together and all BPIs group together separately from the LBPs, the squid LPBs and BPIs do not segregate into these groupings, which is similar in other invertebrates.

### Studies of the symbiosis with *V. fischeri* demonstrate EsBPI2/4 antimicrobial activity and their modulation of the symbiosis.

**FIG 2:**
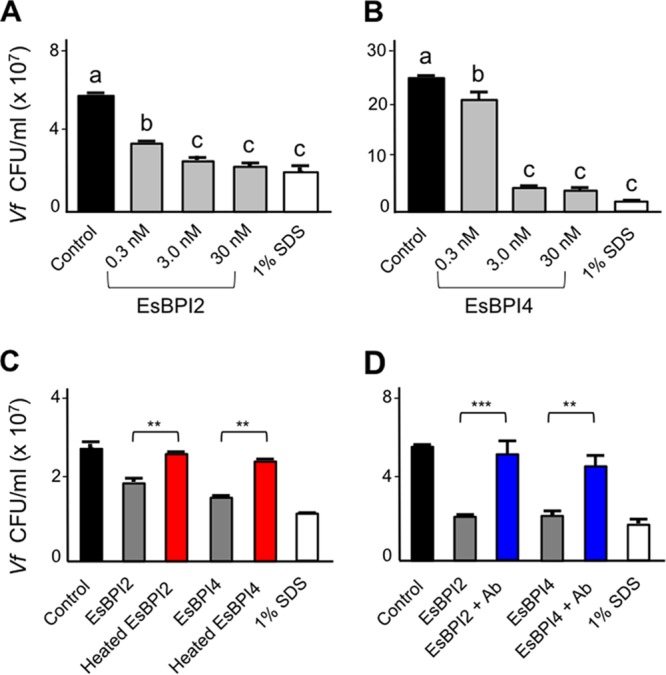
Bactericidal activity of EsBPI2 and EsBPI4 against *V. fischeri*. (A and B) Survival of wild-type *V. fischeri* strain ES114 (*Vf*) following 1-h incubation with different concentrations of EsBPI2 (A) or EsBPI4 (B) compared to incubation with a chemical cell-permeant (SDS). Values are shown as means plus standard errors of the means (sem) (error bars) for three biological replicates, each with three technical replicates. Bars labeled with different letters are statistically significantly different (*P* < 0.001). (C and D) Effect of a pretreatment with either heat (70°C for 10 min; red) (C) or preincubation with the cognate antibody (Ab; blue) (D) on the killing of *V. fischeri* by a 30 nM concentration of either EsBPI2 or EsBPI4 after 1 h of incubation. Values are shown as means plus sem (error bars) for three biological replicates, each with three technical replicates. Pairwise comparisons of viability (CFU/milliliter) for untreated protein/heat-denatured protein (C) and untreated protein/preadsorbed with its cognate antibody protein (D) for each protein were tested by a one-way ANOVA, followed by a Tukey’s multiple-comparison test and are indicated as follows: **, *P* < 0.01; ***, *P* < 0.001. For each protein, heat or antibody treatment resulted in an activity not significantly different from that of the control (black).

EsBPI2 and EsBPI4 were separately purified from squid tissue extracts by immunoprecipitation, using specific antibodies directed against unique internal peptides (Fig. 1A). Although slightly different between the two proteins, both purified EsBPIs had potent antimicrobial activity against *V. fischeri*, significantly reducing the presence of CFU at a concentration as low as 300 pM (Fig. 2A and B); by 30 nM, a concentration where human BPIs are also active (6, 15), both proteins were essentially as effective at killing the cells as 1% SDS. Heat treatment and coincubation with the specific cognate antibody compromised antimicrobial activity of both proteins (Fig. 2C and D).

**FIG 3:**
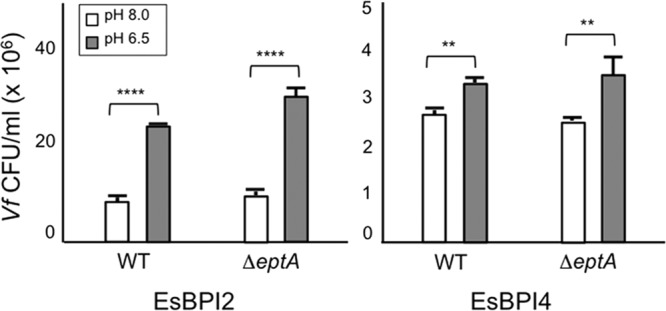
Effect of low-pH pretreatment on *V. fischeri* susceptibility to EsBPI2 and EsBPI4. Survival of either wild-type (WT) or an *eptA* mutant derivative of *V. fischeri* strain ES114 exposed to EsBPI2/4. The two strains were cultured for 3 h in SWT adjusted to either pH 8.0 or 6.5 with 20 mM Tris-HCl prior to a 1-h incubation with one of the EsBPIs. Values are shown as means plus sem (error bars) for three biological replicates, each with three technical replicates. Significant differences in viability (CFU/milliliter) between the two strains with different pH treatments were tested by a two-way ANOVA and are indicated as follows: **, *P* < 0.01; ****, *P* < 0.0001.

During initial contact with the host, *V. fischeri* cells aggregate in acidic, antimicrobial mucus on the light organ surface. These conditions have been hypothesized to “prime” the symbionts to survive subsequent, harsher encounters with such stresses (13, 16). Preliminary transcriptomic and immunocytochemical analyses of EsBPI2/4 suggested that these proteins are abundant in hatchling light organs (see below), and we hypothesized that the acidic conditions experienced by the aggregating symbionts might render *V. fischeri* less susceptible to the antimicrobial activities of EsBPI2/4. We found that pregrowing wild-type *V. fischeri* under mildly acidic conditions (pH 6.5), rather than at the pH of seawater (8.0), i.e., priming the cells, resulted in a significantly greater resistance of cells to the antimicrobial activity of both EsBPI2 and, to a lesser extent, EsBPI4 (Fig. 3). In *Salmonella enterica* serotype Typhimurium, such acid-induced resistance to antimicrobial agents results from activity of the lipid A-modifying enzyme EptA (17). However, this enzyme appears to have no role in the acid-induced resistance to EsBPI2/4 by *V. fischeri*, because an *eptA* mutant responds to acidification indistinguishably from wild-type cells (Fig. 3). Taken together, these data indicate an antimicrobial activity of EsBPI2/4 toward *V. fischeri* that can be reduced by preexposing the bacteria to acidic conditions.

During the onset of symbiosis, expression of genes encoding EsBPI2/4 was low and highly variable. In all animals assayed by quantitative reverse transcription-polymerase chain reaction (qRT-PCR) (Fig. S2), we reproducibly observed higher values in symbiotic animals in expression of the gene encoding EsBPI2, although this difference was not statistically significant (*P* > 0.05). Host exposure to symbiont microbe-associated molecular patterns (MAMPs) that are active in the system, i.e., derivatives of LPS and peptidoglycan, induced a significant, although highly variable, change in EsBPI2 gene expression. For the gene encoding EsBPI4, expression of animals in three of five trials responded strongly to colonization by the symbiont or MAMPs; however, no statistical significance was noted.

10.1128/mBio.00040-17.3 FIG S2  Induction of genes encoding EsBPI2/4 by *V. fischeri* colonization or MAMPs. EsBPI2 (left) and EsBPI4 (right) transcript levels, normalized to the nonsymbiotic condition (Apo), in light organs colonized for 24 h by *V. fischeri* (Es114) or uncolonized light organs of animals exposed for 24 h to 1 µM tracheal cytotoxin (TCT) and 10 ng/ml lipid A from* V. fischeri*. Circles represent the means of two technical replicates performed in each biological condition; gray lines represent the mean of the five biological replicates. Values that were significantly different (*P* < 0.05) for different treatments were tested by a one-way ANOVA, followed by a Tukey’s multiple-comparison test, and are indicated by an asterisk. Download FIG S2, TIF file, 14.1 MB. Copyright © 2017 Chen et al. 2017 Chen et al. This content is distributed under the terms of the Creative Commons Attribution 4.0 International license.

### EsBPI2/4 are abundant in epithelial tissues.

**FIG 4:**
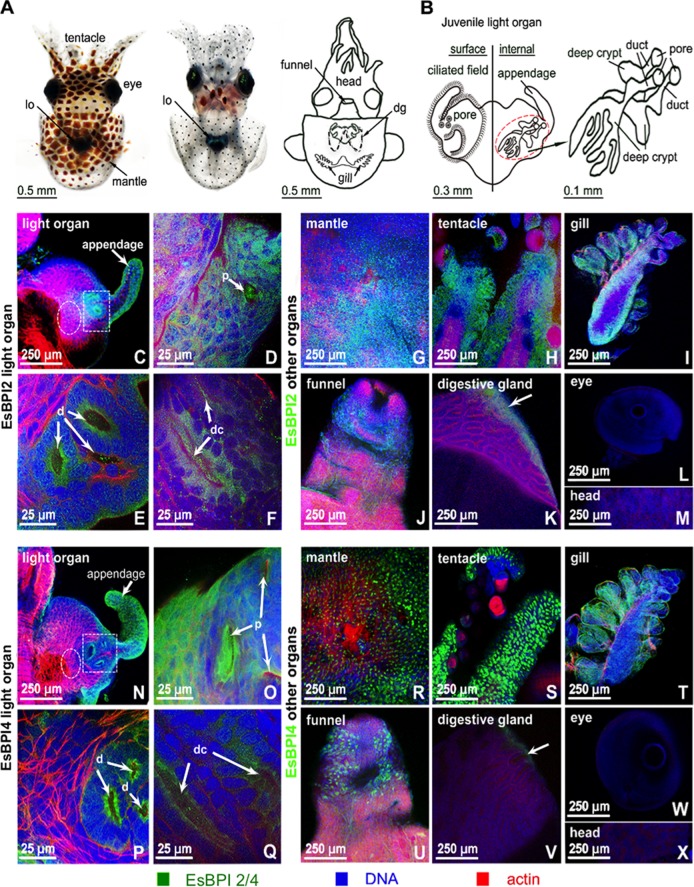
Localization of EsBPI2 and EsBPI4 in juvenile squid tissues. Proteins were probed with rabbit anti-EsBPI2 or anti-EsBPI4 antibodies, which were detected with FITC-conjugated goat anti-rabbit secondary antibody (EsBPI2/4 [green]), and counterstained with rhodamine-phalloidin (actin cytoskeleton [red]) and TOTO-3 (blue) (nuclei). (A) Dorsal and ventral views of the juvenile squid, and diagram to illustrate internal regions depicted in the following panels of the figure. In the diagram, the digestive gland (dg) is shown. lo, light organ. (B) Enlargement of the light organ showing the surface in contact with the environment (left) and the internal structure (right) where symbionts colonize. (Right) Anatomical structures of the three crypts indicated by the dashed red circle. (C to F) EsBPI2 ICC labeling at the top surface of the light organ (C), in the pore (p) (white arrow) (D), in the ducts (d) (white arrows) (area of white dashed box in panel C) (E), and in the deep crypts (dc) (deep crypt lumen indicated by white arrows) (white dashed circle in panel C, which is tissue superficial to the deep crypts) (F). (G to M) Localization of EsBPI2 ICC labeling in other organs examined. The white arrow points to the epithelial edge of the digestive gland. (N to Q) EsBPI4 ICC labeling on the top surface of the light organ (N), in the pores (p) (white arrows) (O), in the ducts (d) (white arrows) (white dashed box area in panel N) (P), and in the deep crypts (dc) (white arrows indicate deep crypt lumen) (white dashed circle in panel N, which is tissue superficial to the deep crypts) (Q). (R to X) Localization of EsBPI4 ICC labeling in other organs examined. The white arrow points to the epithelial edge of the digestive gland.

Both encoded proteins were abundant in epithelial tissues throughout the animal’s body. The proteins labeled strongly in the apical surface of all light organ epithelia (Fig. 4C to F and N to Q). They were also plentiful in the skin, including the mantle surface, i.e., the outer body surface that envelops the organs and tentacles (Fig. 4G, H, R, and S) and other organs, including the gills, funnel, and digestive gland (liver analog) (Fig. 4I to K and T to V). EsBPI2/4 proteins were not observed in the deeper tissues of the head, eyes (Fig. 4L, M, W, and X), and internal musculature (Fig. 4, actin-rich regions shown in red). Thus, antibodies specific to each protein cross-reacted with epithelia exposed directly to environmental seawater. No labeling was observed in control treatments (Fig. S3).

10.1128/mBio.00040-17.4 FIG S3  Controls for immunocytochemistry (ICC) experiments. For a negative control for nonspecific labeling, light organs of juvenile squid were incubated with rabbit IgG under the same conditions as the incubation regimen for EsBPI2/4 antibodies. The DNA counterstain was TOTO-3, and the actin cytoskeleton counterstain was rhodamine-phalloidin. No signal of the secondary antibody (goat anti-rabbit FITC [green]) was observed. Download FIG S3, TIF file, 14.1 MB. Copyright © 2017 Chen et al. 2017 Chen et al. This content is distributed under the terms of the Creative Commons Attribution 4.0 International license.

**FIG 5:**
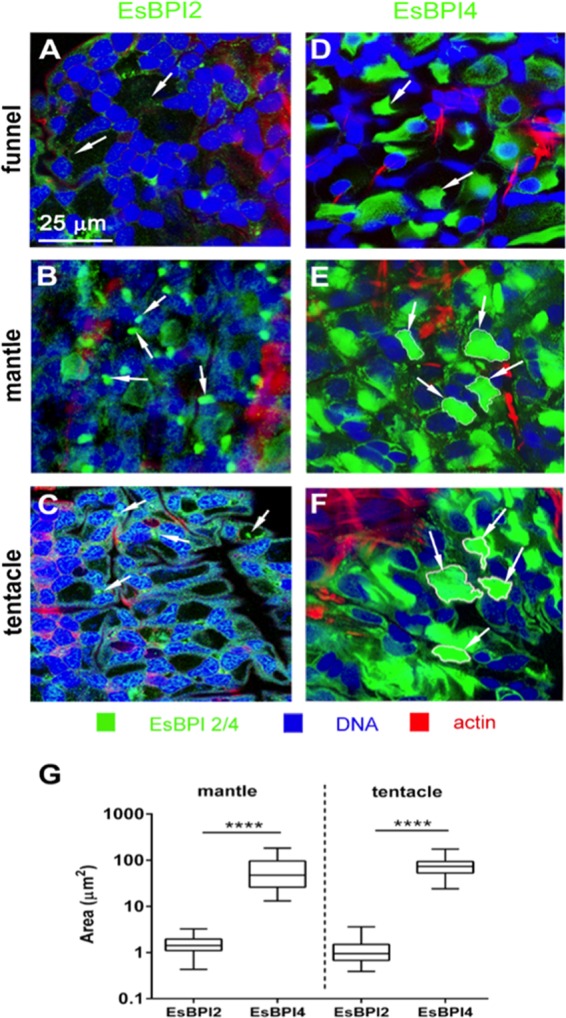
Subcellular localization of EsBPI2 and EsBPI4 by ICC. (See the legend to Fig. 4 for ICC methods.) (A to C) Typical punctate cytosolic ICC labeling of EsBPI2 (white arrows); (D to F) large stores of EsBPI4 in the cytosol (white arrows). (G) Size of labeled areas within cells. The area of cytosolic ICC labeling of EsBPI2 and EsBPI4 was determined by examining 30 regions in each tissue for three different animals. Values that are significantly different (*P* < 0.0001) are indicated by a bar and four asterisks.

The intracellular distributions of EsBPI2 and EsBPI4 in epithelia differed. EsBPI2 occurred as numerous, small puncta throughout the cytosol (Fig. 5A to C). Labeling of EsBPI4 antibodies was also abundant in these tissues but was restricted to the apical regions of the cells, where it occurred in stores 50 to 100 times the area of the EsBPI2 puncta (Fig. 5D to G).

### EsBPI2, but not EsBPI4, is abundant in the hatching organ and bloodstream.

**FIG 6:**
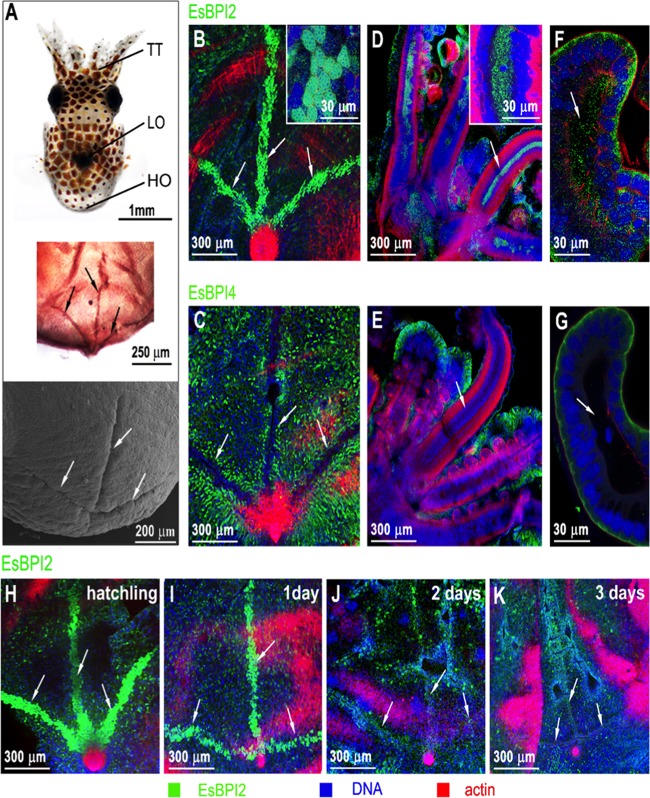
EsBPI2 is unique in the blood and Hoyle organ. (See the legend to Fig. 4 for ICC methods.) (A) Hoyle organ location. (Top) Position of the Hoyle organ (HO) in the body, relative to the tentacles (TT) and light organ (LO). (Middle) Low-magnification light micrograph of the posterior end of the animal showing the trifurcated branching of the HO. (Bottom) Low-magnification scanning electron micrograph, showing the surface view of the HO branches. (B) ICC labeling of EsBPI2 in the HO. (C) Exclusion of EsBPI4 from the HO. (D and E) EsBPI2 ICC labeling of blood sinuses of the tentacles (D) and lack of EsBPI4 ICC labeling of these sites (E). (F and G) EsBPI2 ICC labeling of blood sinuses of the juvenile light-organ superficial appendages (F) and lack of EsBPI4 ICC labeling of these sites (G). Insets in panels B and D show higher-magnification images of ICC labeling of EsBPI2 in the Hoyle organ and blood sinus of the tentacle, respectively. (H to K) The typical loss of the HO in early squid development (29, 31) and concomitant loss of EsBPI2 labeling.

EsBPI2 was abundant in the hatching, or “Hoyle,” organ as well as the blood sinuses of juvenile *E. scolopes*. The Hoyle organ is a trifurcated glandular organ on the posterior, dorsal mantle surface (Fig. 6A). This transient set of tissues contains secretions that enable the juvenile to hatch from the egg. EsBPI2 was abundant within glandular cells of the Hoyle organ (Fig. 6B), while EsBPI4 was excluded from this region (Fig. 6C). In addition, EsBPI2, but not EsBPI4, was detected free within the hemolymph of the blood sinuses (Fig. 6D to G). Whereas the exclusive EsBPI2 labeling occurred in the blood sinuses through 3 days after hatching, EsBPI2 labeling was lost as the Hoyle organ underwent its typical degradation over the first 3 days following hatching (Fig. 6H to K).

### Epithelia interacting with seawater are devoid of colonizing bacteria.

**FIG 7:**
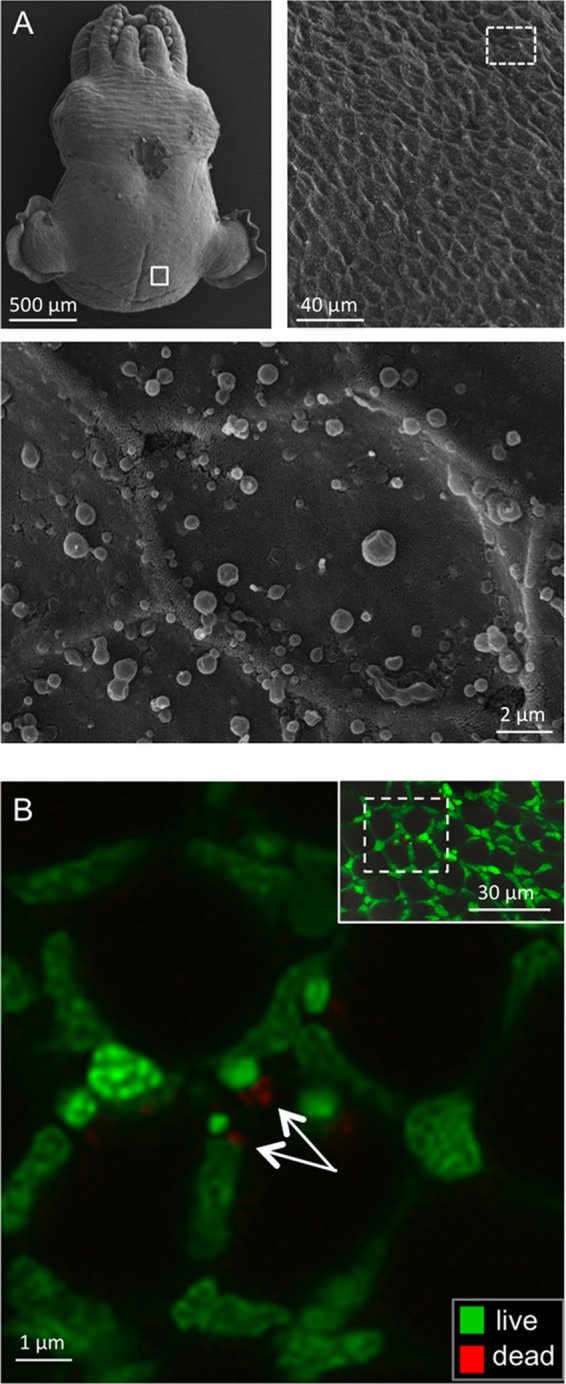
Detection of microbes associating with the outer mantle surface of *E. scolopes*. (A) Scanning electron microscopy (SEM). (Top, right and left) Low-magnification images of the animal showing the region examined for microbes (white boxes). (Bottom) High-magnification image of the epithelial surface (*n* = 4) (the whole skin of the animal was examined) with no microbes detected. (The irregular, round globules on the surface are mucous secretions from the skin.) (B) Confocal micrographs of a live, anesthetized animal that had been washed with filter-sterilized seawater three times for 5 min each time and then labeled for detection of live (green) or dead (red) bacterial cells. Images were taken in the same surface region as for SEM. (Inset, top right) Low-magnification view to show region (box outlined with white dashed line) examined at higher magnification. White arrows indicate possible vestiges of dead bacterial cells (*n* = 5).

Because EsBPI2 and EsBPI4 were abundant in epithelia interacting with environmental seawater, we examined the skin for association with bacterioplankton. No bacteria were detected by scanning electron microscopy (SEM) on the mantle or gill surfaces (Fig. 7A and Fig. S4A and S4B). Because bacteria may be lost during SEM sample preparation, we also analyzed the epithelia of living, anesthetized animals for associating bacteria by using fluorochromes that label live and dead bacterial cells (LIVE/DEAD BacLight bacterial viability kit; Thermo Fisher Scientific). Using cultured *V. fischeri* cells, we first confirmed that the antimicrobial activity of EsBPI2/4 could be visualized with the fluorochromes. Confocal microscopy of live-animal surfaces revealed the occasional dead cell, but no live bacteria were detected on the epithelial surfaces (Fig. 7B and Fig. S4C).

10.1128/mBio.00040-17.5 FIG S4  Absence of bacteria on host gill surfaces. (A) Low-magnification SEM showing the structure of the gills. The white box in panel A shows the location of the image in panel B. (B) Higher-magnification SEM showing gill surface at the scale of bacterial cells. (C) Confocal live-dead labeling. Live labeling is shown in green, and dead labeling is shown in red. Host nuclei label green. (Inset) The white box shows the region where a high-magnification confocal microscopic view was acquired. The small amount of red labeling (white arrow) may be vestiges of a dead bacterial cell. (*n* = 5 for both treatments.) Download FIG S4, TIF file, 14.1 MB. Copyright © 2017 Chen et al. 2017 Chen et al. This content is distributed under the terms of the Creative Commons Attribution 4.0 International license.

## DISCUSSION

The two BPIs characterized in this study, EsBPI2 and EsBPI4, exhibit typical biochemical, structural, and antimicrobial characteristics of animal BPIs. The spatial and temporal patterns of their occurrence across the eight organs studied in the hatchling animal suggest that EsBPI2/4 proteins modulate host-microbial interactions in both external and internal regions of the body. They share localization to the mucosal epithelia. As such, they are similar to the BPIs that are secreted along mucosal surfaces in terrestrial vertebrates, such as birds (5) and mammals (18).

The presence of both EsBPI2 and EsBPI4 in the epithelia of the juvenile light organ (Fig. 4C to F and N to Q) suggests roles for these proteins in specificity and control of symbiont populations. These proteins colocalize with other antimicrobials, such as nitric oxide (19), EsPGRP2 (*E. scolopes* peptidoglycan recognition protein 2) (20), galaxin (21), and hemocyanin (22) that are secreted with low-pH mucous stores from the superficial ciliated fields of the light organ within minutes following hatching. Resolution of *V. fischeri* as the sole colonizer of the *E. scolopes* light organ begins while the bacteria are still outside tissues but within the mucous matrix along this ciliated field. Our present model of this process posits that the biomechanical forces of the ciliated field work in concert with an antimicrobial “cocktail,” which would include the BPIs, to render *V. fischeri* the competitive dominant in this microenvironment. Our finding that *V. fischeri* can be primed by low pH to be more resistant to the antimicrobial activity of the BPIs provides a mechanism by which the bacterial cells can be prepared for constant presentation of BPIs by the internal epithelial surfaces of the organ. Although in other systems a low-pH environment induces resistance to antimicrobials through the lipid A-modifying enzyme EptA (17, 23), in *V. fischeri* this activity was not required for the acid-primed resistance to BPIs. As such, the mechanism underlying this priming remains to be discovered.

Immunocytochemistry (ICC) localized both BPIs to the epithelia of the crypts of both nonsymbiotic and symbiotic animals. As such, the bacteria would experience the activity of these proteins both upon entering the crypt space and persistently. The involvement of antimicrobials in the modulation of symbionts has been reported for mammals (e.g., RegIII [24]), other invertebrates (e.g., arminins in *Hydra* spp. [25]), and the crypts of *E. scolopes* (e.g., EsPGRP2 [20] and EsGal1 [21]). The abundance of EsBPI2 and EsBPI4 in all epithelia and the paucity of environmental bacteria at these sites, including *V. fischeri*, suggest that these proteins not only modulate host interactions with the symbiont in the light organ, but these proteins may also be involved in protecting the surface from fouling by *V. fischeri* and other environmental bacteria. Because juvenile host animals expel about 10^6^ *V. fischeri* cells daily, the symbionts represent an abundant source of potential colonizers. Whether EsBPI2 and EsBPI4 are the antimicrobials responsible for the absence of bacteria other than *V. fischeri* on nonsymbiotic host tissues and whether these proteins participate in controlling fouling of epithelial surfaces in other mollusks remain to be studied.

The different subcellular localization in epithelia (Fig. 4 and 5) suggests different behaviors in the cells, but an understanding of the significance of this difference will require further research. The presence of EsBPI2 in the blood and hatching gland, or Hoyle organ (26), and the exclusion of EsBPI4 from those sites provides evidence for a role for EsBPI2 in processes other than protection of epithelia. EsBPI2 in the blood likely acts similarly to BPIs in mammals, where they are a line of defense against sepsis (27, 28). Localization of EsBPI2 in the Hoyle organ is a new finding, although the Hoyle organ of cephalopods has been studied for more than 120 years (29). The involvement of a BPI in defense of the eggs in the snail *Biomphalaria glabrata* provides precedence for the protective activity of BPIs during development in mollusks (1, 30). At hatching, the juvenile positions its posterior at the surface of the egg capsule and releases enzymes from the Hoyle organ epithelium that digest a hole in the capsule through which the juvenile will escape the egg. Many detailed characterizations of the structure and function of Hoyle organs have been reported (26, 31–35), and basic proteins were identified in the composition of Hoyle organ secretions, but their identity had not been determined (30). The disappearance of EsBPI2 in this area occurs over the first 3 days following hatching concomitantly with the typical developmental loss of the Hoyle organ (36, 37).

This study provides a window into how different antimicrobial proteins can serve multiple functions in the relationships of an animal with its mutualistic symbionts, and possibly with potential pathogens in its environment. The diverse biochemical strategies used by soft-bodied marine invertebrates in their interactions with ambient bacteria present a rich frontier. The broad occurrence of BPIs across the animal kingdom suggests a role for these proteins in such activities.

## MATERIALS AND METHODS

### Sequence analysis, structural modeling, and phylogenetic tree construction.

We obtained full-length sequence of the EsBPI4 gene by rapid amplification of cDNA ends (RACE) from partial sequences in a shotgun-sequenced transcriptome (RNAseq) (13); the full-length sequence of the EsBPI2 gene had already been determined (2). We aligned the derived amino acid sequences using default parameter BLASTP. Using the CLC Sequence Viewer software (CLC Bio, Qiagen), we generated the alignment graph of EsBPI2 and EsBPI4 sequences with one another and with EsLBP1 and human BPI (hBPI). BLAST software was used in homology searches (38). We obtained predictions of signal peptides and domains with the SignalP (http://www.cbs.dtu.dk/services/SignalP/) and SMART (39) software, respectively. To compute the theoretical isoelectric point (pI) and molecular weight (Mw), we used the Compute pI/Mw tool (40, 41). We created the three-dimensional (3-D) models of EsBPI2 and EsBPI4 with SWISS-MODEL (42–45) using the 1.70-Å and 2.40-Å crystal structures of human BPI (SWISS-MODEL template identifier [ID] 1ewf.1.A and 1bp1.1.A) as a template, respectively. We chose the model with the best QMEAN4 score (46).

We generated an unrooted phylogenetic tree of LBP/BPIs by searching the NCBI database using default parameter BLASTP. Only full-length sequences that included both BPI domains were used in this tree. In addition, we selected only those sequences with E values of <10^−30^ for at least one of the four EsLBP/BPI sequences. If a given organism had sequences with both LBP and BPI activity, both sequences were included in our analysis. With sequences assembled, we generated an alignment with CLC Sequence Viewer software (CLC Bio, Qiagen), applied Gblocks to eliminate nonconserved amino acid stretches (http://www.phylogeny.fr), and followed with maximum-likelihood phylogenetic analysis using the software program PhyML 3.0 (47), assuming a Whelan and Goldman (WAG) model (accession numbers of proteins used in the tree in Table S2 in the supplemental material). We used the approximate likelihood ratio test (aLRT) scores to assess robustness of branching patterns of molecular phylogenies. Such scores correlate with maximum likelihood bootstrap scores but require less computational time (48).

10.1128/mBio.00040-17.7 TABLE S2  Proteins used to construct the LBP/BPI phylogenetic tree in Fig. 1C. Download TABLE S2, DOCX file, 0.02 MB. Copyright © 2017 Chen et al. 2017 Chen et al. This content is distributed under the terms of the Creative Commons Attribution 4.0 International license.

To test for positive selection on the LBP/BPIs, we used the protein alignment of EsLBP1 and EsBPI-2, -3, and -4 to guide the nucleotide alignment (using PAL2NAL). We computed the ratio of nonsynonymous to synonymous mutations (*dN*/*dS* ratio) using PAML (codeML) and HyPhy. Both produced similar results (*dN*/*dS* ratio of <1), indicating fewer nonsynonymous mutations (*dN*) than synonymous mutations (*dS*).

### Colonization of the host with *V. fischeri*.

We captured/maintained adult host animals and colonized juvenile animals with *V. fischeri* as previously described (for a review, see reference 49). We used newly hatched animals, as well as symbiotic and nonsymbiotic juveniles at 24, 48, and 72 h after hatching in immunocytochemistry (ICC) experiments, and 24-h nonsymbiotic/symbiotic animals for light organ RNA extraction in quantitative reverse transcription-PCR (qRT-PCR) experiments. For purification of EsBPIs for antimicrobial assays, we used tissue extracts from uncolonized juvenile animals. All animal experiments conform to relevant regulatory standards established by the University of Wisconsin—Madison and the University of Hawaii at Manoa.

### Protein purification by immunoprecipitation.

We extracted total soluble protein from the skin of juvenile *E. scolopes*, where we had evidence for both EsBPI2 and EsBPI4 proteins in abundance. Briefly, we dissected tissues from frozen juvenile animals and homogenized them in filtered and autoclaved 70% seawater to which we added Complete protease inhibitor cocktail (Sigma-Aldrich). We recovered the aqueous soluble fraction by centrifuging at 15,000 × *g* for 20 min at 4°C and reserving the supernatant.

Rabbit polyclonal antibodies (Ab) were raised against synthetic peptide antigens (GenScript) designed from divergent regions of EsBPI2 and EsBPI4 (Fig. 1A); the antibody IgG fractions were purified by affinity chromatography from the serum. The two regions contain low similarity to other EsLBP/BPI protein sequences and other protein sequences predicted in the *E. scolopes* transcriptomic databases. We used these antibodies to purify the proteins by immunoprecipitation (Dynabeads protein A immunoprecipitation kit; Thermo Fisher Scientific) following the manufacturer’s protocol. To cross-link the antibody to the Dynabeads, we used BS3 (sulfo-DSS) crosslinker (Thermo Fisher Scientific). For protein analysis, we used standard sodium dodecyl sulfate-polyacrylamide gel electrophoresis (SDS-PAGE) (NuPAGE Novex 12% Bis-Tris protein gels).

### EsBPI2/4 antimicrobial activity assays.

To determine the effect of EsBPI2 and EsBPI4 on the viability of wild-type *V. fischeri* strain ES114 (50), we grew cells aerobically to mid-log phase at 28°C in SWT medium, which contains 0.5% (wt/vol) Bacto tryptone, 0.3% (wt/vol) yeast extract, and 0.3% (vol/vol) glycerol, in 70% seawater. We then diluted cultures into SWT medium to an optical density at 600 nm (OD_600_) of between 0.02 and 0.04, and incubated aliquots for 1 h with shaking at 28°C, either with or without the following additions: (i) EsBPI2 or EsBPI4, (ii) heat-denatured (70°C for 10 min) EsBPI2 or EsBPI4, or (iii) EsBPI2 or EsBPI4 preadsorbed with its cognate antibody (see above) for 30 min at room temperature before adding to the bacterial cultures. After the incubation, we serially diluted the samples into sterile 70% seawater (SSW) and plated 10-µl aliquots of the 10^−2^ to 10^−6^ dilutions onto SWT medium containing 1.5% (wt/vol) agar, using the drop plate method (51). Specifically, we inoculated 3 drops of each dilution onto the plate (3 replicates). After a 24-h incubation at 28°C, we counted the CFU of the lowest dilution with discernible colonies. We analyzed the data in the GraphPad Prism software package (version 6.02). Treatment-survival data are presented as CFU/milliliter. To determine whether the acid-induced, EptA-catalyzed, modification of the lipid A portion of LPS (17) provided protection against EsBPI2/4, we cultured *V. fischeri* strain ES114 and its *eptA* deletion derivative for 3 h in SWT medium that had been adjusted to either pH 8.0 or 6.5 with 20 mM Tris-HCl. Following this pH pretreatment, we incubated the diluted cultures with EsBPI2 or EsBPI4 as described above, and determined the viable number of CFU remaining per milliliter.

### Immunocytochemistry.

We performed immunocytochemistry (ICC) experiments using a protocol modified from previous studies (20, 52), incubating the samples in either anti-EsBPI2 or anti-EsBPI4 Ab at 1:100 (vol/vol) dilution for 14 days at 4°C. For control experiments, we incubated animals in the same concentration of purified rabbit IgG diluted in the blocking solution. We applied a 1:25 dilution of fluorescein isothiocyanate (FITC)-conjugated goat anti-rabbit IgG (Jackson ImmunoResearch Laboratories) in blocking solution as the secondary antibody for 18 h at 4°C. We then incubated the samples at 4°C for 18 h in a 1:40 dilution of rhodamine-phalloidin (Invitrogen), which served as a counterstain for actin cytoskeleton, and at room temperature for 30 min in a 1:625 dilution of TOTO3 (Invitrogen), which labeled nuclear DNA; both stains were diluted in the blocking solution.

### Scanning electron microscopy.

For scanning electron microscopy (SEM) analyses, we transferred whole juveniles into marine fixative consisting of 2.5% paraformaldehyde and 2% glutaraldehyde in filtered seawater, and incubated them at 4°C for 18 h. We then rinsed the samples twice with 0.1 M cacodylate buffer (pH 7.4) containing 0.35 M sucrose for a total of 30 min. The samples were postfixed with 1% osmium tetroxide in the cacodylate buffer for 1 h, dehydrated in a graded ethanol series, critical point dried with liquid CO_2_, sputter coated with gold particles, and examined with a Hitachi S-4800 field emission scanning electron microscope.

### Labeling bacteria in living squid.

To assay for persistence on the epithelia, we incubated newly hatched animals (*n* = 5) in natural unfiltered seawater for 3 h. We then transferred the hatchlings through three rinses of filtered (0.22-µm) seawater for 10 to 15 min each rinse; the presence and viability of bacteria on the skin of juvenile squid was determined using the LIVE/DEAD BacLight bacterial viability kit as described above.

### Statistical analysis.

For quantitative analysis of experimental data, we used the GraphPad Prism software package (version 6.02). We made comparisons between treatments (with/without protein, untreated protein/ heat-denatured protein, untreated protein/preadsorbed with its cognate antibody protein) with variance analysis to test whether EsBPI2/4 have any effect on the viability of the investigated bacteria. The viability differences between them were tested by a one-way analysis of variance (ANOVA), followed by a Tukey’s multiple-comparison test. We used two-way ANOVA to test whether EsBPI2/4 have any effect on the viability of the two strains with different pH treatments. We also compared the area of cytosolic ICC labeling of the proteins on the skin and the regulation of EsBPI2/4 genes in the light organ under different experimental conditions. Both the area and regulation differences were tested by a one-way ANOVA, followed by a Tukey’s multiple-comparison test. A *P* value of <0.05 was considered statistically significant.

### Accession number(s).

The sequence data for EsBPI4 gene has been submitted to GenBank database under accession number KU951902. The accession number for EsBPI2 (formerly EsLBP2) has already been published (2).[Supplementary-material textS1][Supplementary-material tabS3]

10.1128/mBio.00040-17.1 TEXT S1  Supplemental methods, references, and figure legends. Download TEXT S1, DOCX file, 0.03 MB. Copyright © 2017 Chen et al. 2017 Chen et al. This content is distributed under the terms of the Creative Commons Attribution 4.0 International license.

10.1128/mBio.00040-17.8 TABLE S3  Primers and fragment size in qRT-PCR experiments. Download TABLE S3, DOCX file, 0.02 MB. Copyright © 2017 Chen et al. 2017 Chen et al. This content is distributed under the terms of the Creative Commons Attribution 4.0 International license.
