# Supplementary figures and images for: Bactericidal Permeability-Increasing Proteins Shape Host-Microbe Interactions
Source: mBio. 2017 Apr 4;8(2):e00040-17. doi: 10.1128/mBio.00040-17 (PMC5380838; doi:10.1128/mBio.00040-17)

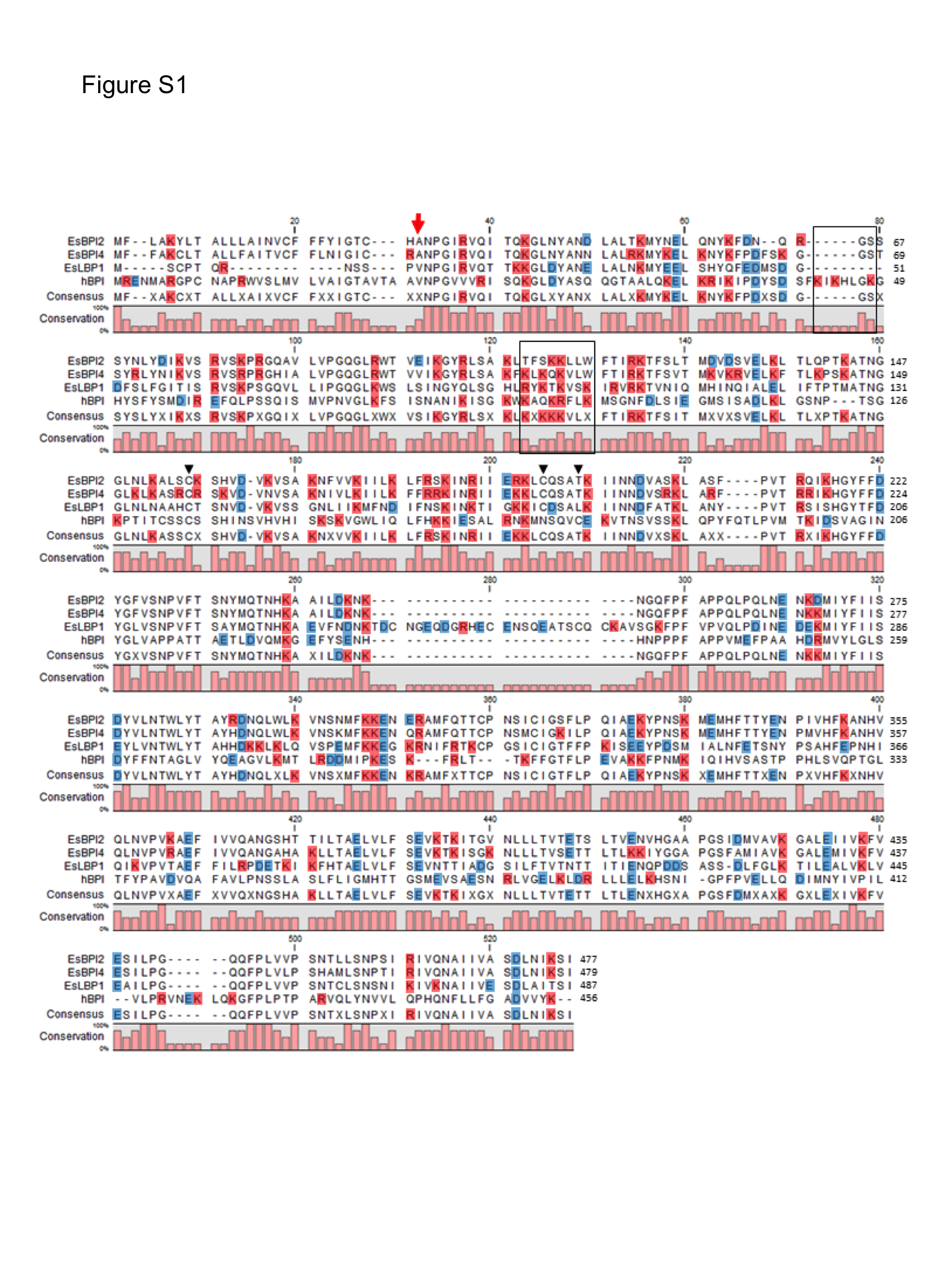

Supplement: FIG S1 [file mbo002173253sf1.tif]

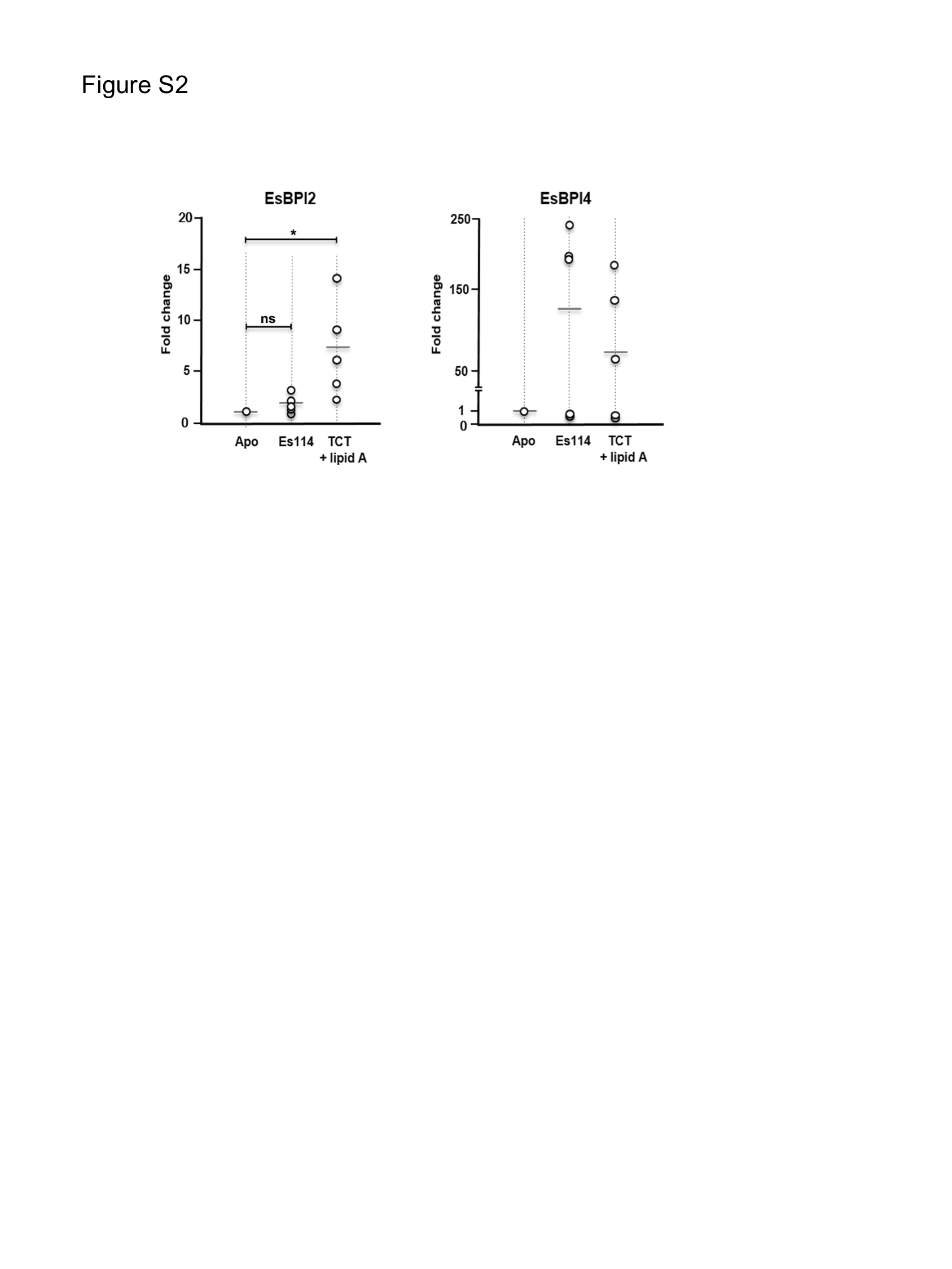

Supplement: FIG S2 [file mbo002173253sf2.tif]

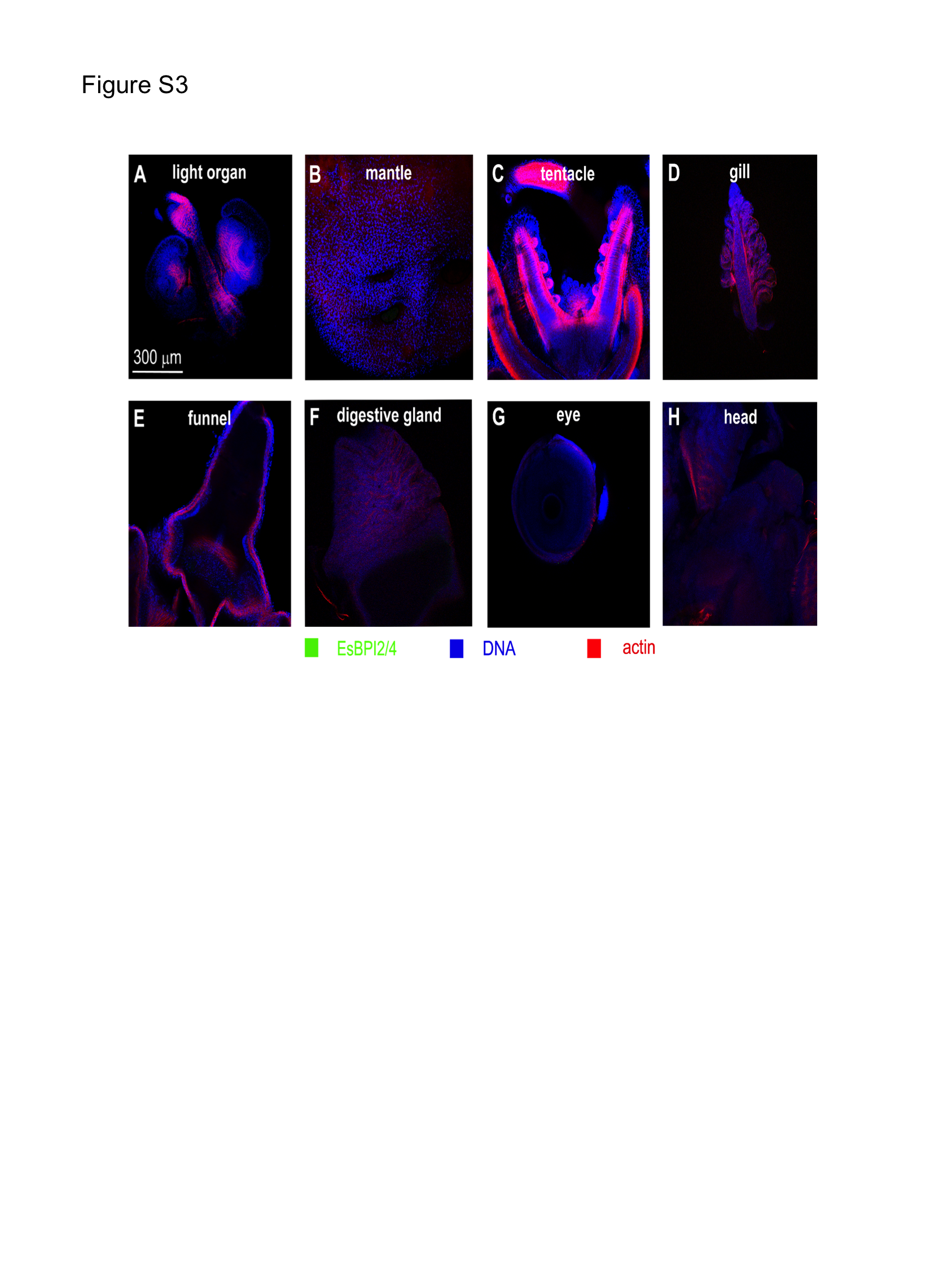

Supplement: FIG S3 [file mbo002173253sf3.tif]

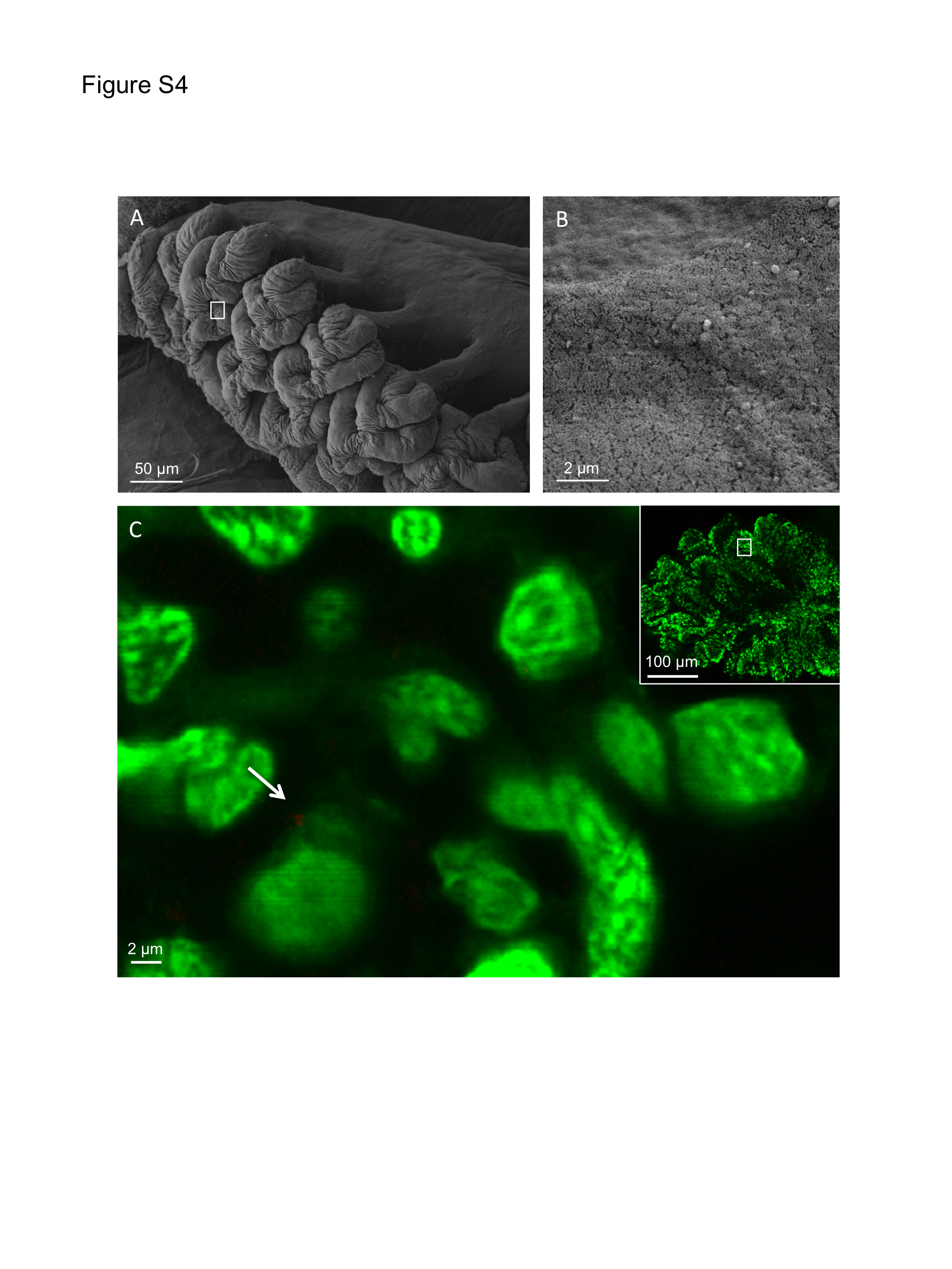

Supplement: FIG S4 [file mbo002173253sf4.tif]
