# Supplementary material for: Bactericidal Permeability-Increasing Proteins Shape Host-Microbe Interactions
Source: mBio. 2017 Apr 4;8(2):e00040-17. doi: 10.1128/mBio.00040-17 (PMC5380838; doi:10.1128/mBio.00040-17)
Supplement: TABLE S1 [file mbo002173253st1.docx]

**Table S1.** Protein sequence alignments and accession numbers.

| Sequence alignment | Identities | Accession numbers |
| --- | --- | --- |
| EsBPI2 vs. hBPI | 91/336(27%) | AEL03861.1 (EsBPI2) AAA51841.1 (hBPI) |
| EsBPI2 vs. EsLBP1 | 251/477(53%) | AEL03860.1 (EsLBP1) |
| EsBPI2 vs. EsBPI3 | 87/353(25%) | AEL03862.1 (EsBPI3) |
| EsBPI2 vs. EsBPI4 | 393/479(82%) | AOS59889.1 (EsBPI4) |
