# Supplementary material for: Bactericidal Permeability-Increasing Proteins Shape Host-Microbe Interactions
Source: mBio. 2017 Apr 4;8(2):e00040-17. doi: 10.1128/mBio.00040-17 (PMC5380838; doi:10.1128/mBio.00040-17)
Supplement: TABLE S2 [file mbo002173253st2.docx]

**Table S2.** Proteins used to construct the LBP/BPI phylogenetic tree in Fig. 1C.

| Species | Protein name | Accession numbers |
| --- | --- | --- |
| *Euprymna scolopes* | EsLBP1 | AEL03860.1 |
| *Euprymna scolopes* | EsBPI2 | AEL03861.1 |
| *Euprymna scolopes* | EsBPI3 | AEL03862.1 |
| *Euprymna scolopes* | EsBPI4 | AOS59889.1 |
| *Octopus_bimaculoides* | Octopus (Ob)_LBP | XP 014783324.1 |
| *Biomphalaria_glabrata* | Fresh water snail (Bg)_BPI | XP 013088376.1 |
| *Biomphalaria_glabrata* | Fresh water snail (Bg)_LBP | XP 013070671.1 |
| *Strongylocentrotus_purpuratus* | Sea urchin (Sp)_BPI | XP 794672.3 |
| *Apostichopus_japonicus* | Sea cucumber (Aj)_LBP | AKH45418.1 |
| *Eisenia_andrei* | Earthworm (Ea)_BPI | AFI44048.1 |
| *Canis_lupus_familiaris* | Dog (Clf)_LBP | XP 542993.3 |
| *Canis_lupus_familiaris* | Dog (Clf)_BPI | XP 534417.2 |
| *Mustela_putorius_furo* | Weasel (Mpf)_LBP | XP 004746502.1 |
| *Mustela_putorius_furo* | Weasel (Mpf)_BPI | XP 012908774.1 |
| *Equus_przewalskii* | Horse (Ep)_LBP | XP 008515639.1 |
| *Equus_przewalskii* | Horse (Ep)_BPI | XP 008515640.1 |
| *Ceratotherium_simum_simum* | Rhinoceros (Cs)_BPI | XP 004430623.1 |
| *Ceratotherium_simum_simum* | Rhinoceros (Cs)_LBP | XP 004430467.1 |
| *Elephantulus_edwardii* | Elephant (Ee)_BPI | XP 006881583.1 |
| *Elephantulus_edwardii* | Elephant (Ee)_LBP | XP 006881773.1 |
| *Ictidomys_tridecemlineatus* | Ground squirrel (It)_LBP | XP 013216002.1 |
| *Ictidomys_tridecemlineatus* | Ground squirrel (lt)_BPI | XP 005329985.1 |
| *Tupaia_chinensis* | Shrew (Tc)_BPI | XP 006144189.1 |
| *Tupaia_chinensis* | Shrew (Tc)_LBP | XP 006144191.1 |
