# Supplementary material for: Bactericidal Permeability-Increasing Proteins Shape Host-Microbe Interactions
Source: mBio. 2017 Apr 4;8(2):e00040-17. doi: 10.1128/mBio.00040-17 (PMC5380838; doi:10.1128/mBio.00040-17)
Supplement: TEXT S1 [file mbo002173253s1.docx]

**SUPPLEMENTAL INFORMATION FOR:**

**Bactericidal/permeability-increasing Proteins (BPIs) Shape Host-microbe Interactions**

**SUPPLEMENTAL METHODS**

**RNA extraction.** We stabilized tissues dissected from the collected samples at -80°C in RNAlater (Ambion, Austin, TX) until RNA extraction. Using the RNeasy Mini Kit (Qiagen) according to the manufacturer's instructions, we extracted RNA from samples for molecular analyses. We analyzed the extracts by gel electrophoresis to assess quality of the extracted RNA and estimated its quantity spectrophotometrically using a NanoDrop ND1000.

**CDNA preparation.** For the extracted RNA, we removed contaminating DNA using the Turbo DNA Free kit (Ambion, Austin, TX). We performed cDNA synthesis with SMART MMLV Reverse Transcriptase (Clontech, Mountain View, CA) according to the manufacturer’s instructions, using 500 ng of total RNA for each reverse transcription (RT) reaction. Each reaction sample was diluted 6-fold in nuclease-free water and stored for later use, short-term at 4^o^C and long term at -20°C.

**Quantitative Reverse Transcription-Polymerase Chain Reaction (qRT-PCR).** We stabilized dissected light organs in RNALater for qRT-PCR (n = 30/replicate; 5 biological replicates/condition) and stored them at -80°C until RNA extraction. We performed RNA extraction and cDNA preparation as described above. As described previously (1), qRT-PCR experiments were conducted in accordance with MIQE guidelines (2) using a CFX Connect Real-Time System (BioRad, Hercules, CA) as follows: 3 min at 95°C; 40 cycles of 10 s at 95°C, 10 s at 60°C, and 15 s at 68°C; and 10 s for 95°C followed by a temperature gradient from 65 to 95°C at 5°C intervals, 5 s per interval. QRT-PCR for each gene was performed with five biological replicates and two technical replicates per condition. The sequences of the gene-specific primers used here are provided in Table S3. The reaction mixture for qRT-PCR consisted of 5 μL of the LC480 SYBR-Green master mix (Roche), 0.5 μL of each primer (10 mM), and 4 μL of a 1/6 dilution of the cDNA reaction. The efficiencies of all qRT-PCR primer sets were between 98% and 105%. We used the comparative quantification cycle (ΔΔCq) method to determine expression levels (3) (Pfaffl, 2001). We normalized the gene expression levels of EsBPI2 and EsBPI4 to the mean expression levels of two genes, ribosomal 40S and serine HMT.

**SUPPLEMENTAL REFRENCES**

1. **Peyer SM, Pankey MS, Oakley TH, McFall-Ngai MJ.** 2014. Eye-specification genes in the bacterial light organ of the bobtail squid Euprymna scolopes, and their expression in response to symbiont cues. Mech Dev **131:**111-126.

2. **Bustin SA, Benes V, Garson JA, Hellemans J, Huggett J, Kubista M, Mueller R, Nolan T, Pfaffl MW, Shipley GL, Vandesompele J, Wittwer CT.** 2009. The MIQE guidelines: minimum information for publication of quantitative real-time PCR experiments. Clin Chem **55:**611-622.

3. **Pfaffl MW.** 2001. A new mathematical model for relative quantification in real-time RT-PCR. Nucleic Acids Res **29:**e45-e45.

4. **Beamer LJ, Carroll SF, Eisenberg D.** 1998. The BPI/LBP family of proteins: a structural analysis of conserved regions. Protein Sci **7:**906-914.

**SUPPLEMENTAL FIGURE LEGENDS**

**Fig. S1.** Alignment of full-length sequences of the EsBPI2 and EsBPI4 with one another and with EsLBP1 and hBPI (human BPI). Solid boxes - Region corresponding to: i) residues 42-48 of human BPI, including conserved basic residues at positions 42 and 48 of human BPI; and ii) region corresponding to residues 92-99 of hBPI, including conserved basic residues at positions 92, 95 and 99 of hBPI (4). Black arrowheads, cysteines thought to correspond to conserved disulfide bond of LBP/BPI proteins. Red arrow, typical starting point for numbering of hBPI, which excludes the signal sequence (4). Red shading: basic residues (Arg, Lys). Blue shading: acidic residues (Glu, Asp).

**Fig. S2.** Induction of genes encoding EsBPI2/4 by *V. fischeri* colonization or MAMPs. EsBPI2 (left) and EsBPI4 (right) transcript levels, normalized to the nonsymbiotic condition (Apo), in light organs colonized for 24 h by *V. fischeri* (Es114) or uncolonized light organs of animals exposed for 24 h to 1 µM tracheal cytotoxin (TCT) and10 ng/mL lipid A from *V. fischeri* . Circles, the mean of two technical replicates performed in each biological condition; gray lines, the mean of the five biological replicates. Regulation significant differences (* = p < 0.05) between different treatments were tested by a one-way ANOVA followed by a Tukey’s multiple comparisons test.

**Fig. S3.** Controls for immunocytochemistry (ICC) experiments. As a negative control for non-specific labeling, light organs of juvenile squid were incubated with rabbit IgG under the same conditions as the incubation regimen for EsBPI2/4 antibodies. DNA counterstain, TOTO-3; actin-cytoskeleton counterstain, rhodamine-phalloidin. No signal of the secondary antibody (goat anti-rabbit FITC; green) was observed.

**Fig. S4.** Absence of bacteria on host gill surfaces. (A) Low magnification SEM showing the structure of the gills. White box, location of image in B. (B) Higher magnification SEM showing gill surface at the scale of bacterial cells. (C) Confocal live (green)-dead (red) labeling. Host nuclei label green. Inset, white box, region where a high magnification confocal was acquired. The small amount of red labeling (white arrow) may be vestiges of a dead bacterial cell. [N = 5 for both treatments]
