# Supplementary material for: Bactericidal Permeability-Increasing Proteins Shape Host-Microbe Interactions
Source: mBio. 2017 Apr 4;8(2):e00040-17. doi: 10.1128/mBio.00040-17 (PMC5380838; doi:10.1128/mBio.00040-17)
Supplement: TABLE S3 [file mbo002173253st3.docx]

**Table S3:** Primers and fragment size in qRT-PCR experiments.

| Gene | Primer name | Primer sequence | Fragment size |
| --- | --- | --- | --- |
| EsBPI2 | EsBPI2qF | ACCACAAATTGCGGAGAAGT | 139 bp |
| EsBPI2 | EsBPI2qR | GGCTTGAACCACGATGAACT |  |
| EsBPI4 | EsBPI4qF | CGTGTC CCA ATTCTATGT GC | 174 bp |
| EsBPI4 | EsBPI4qR | GGCTTG AACCAC GATGAA CT |  |
| Serine HMT | SerHMTqF | GTCCTGGTGACAAGAGTGCAATGA | 103 bp |
| Serine HMT | SerHMTqR | TTCCAGCAGAAAGGCACGATAGGT |  |
| Ribosomal 40S | 40SF | AATCTCGGCGTCCTTGAGAA | 188 bp |
| Ribosomal 40S | 40SR | GCATCAATTGCACGACGAGT |  |
